# A Non-ossifying Fibroma and a Stress Fracture of the Femur Mimicking Bone Malignancy in a Child

**DOI:** 10.7759/cureus.7652

**Published:** 2020-04-12

**Authors:** Svetoslav A Slavchev, Georgi P Georgiev

**Affiliations:** 1 Orthopaedics and Traumatology, Medical University of Sofia, Sofia, BGR; 2 Orthopaedics and Traumatology, University Hospital Queen Giovanna, Sofia, BGR

**Keywords:** non-ossifying fibroma, stress fracture, femur, differential diagnosis, radiography, malignancy, children, bone tumors

## Abstract

Herein, we report a rare case of a stress fracture through a pre-existing non-ossifying fibroma (NOF) of the femur of a 12-year-old child that raised suspicion of a malignancy. Although NOFs are very frequent, in the vast majority of cases, they are completely asymptomatic. When encountered in a painful area, especially if combined with atypical radiographic features, they may mimic a malignancy. We discuss the radiographic findings, the differential diagnosis, and the relevant points of the patient's history that helped to establish the diagnosis.

## Introduction

Benign and non-neoplastic conditions can mimic malignant bone tumors in imaging studies. Usually, each of them separately does not pose a significant diagnostic problem, like a non-ossifying fibroma (NOF) and a stress fracture. When they exist simultaneously, however, potential diagnostic difficulties can increase, especially when faced by a physician who is less experienced in musculoskeletal pathology.

In those cases, the correct diagnosis could be established after a careful study of the patients’ history and clinical status, together with different imaging modalities. After precise evaluation, a correct diagnosis and plan for treatment could be made [1,2]. Stress fractures in children and adolescents are known to have been mistaken for bone sarcomas [3]. NOF is a benign lesion that arises in the metaphyses of long bones, commonly around the knee, in children and adolescents with an estimated incidence of 20%-30% [1,4]. In the immature skeleton, the stress fractures are due to abnormal muscular activity on bones and usually are localized in the proximal tibia followed by the distal femur and fibula [3]. The stress fractures and NOF have been reported to affect similar locations in long bones. A pre-existing NOF can weaken the bone and thus predispose to fracture [1].

## Case presentation

**Figure 1:**
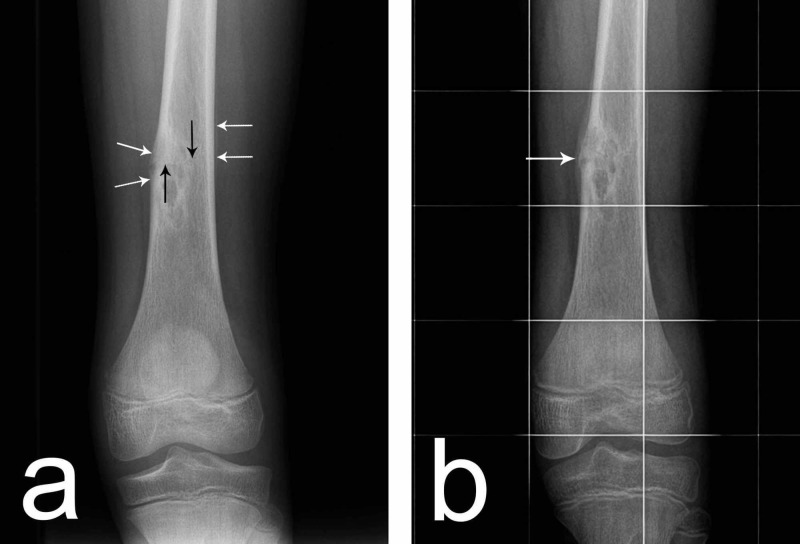
(a) Periosteal reaction (white arrows) and fracture line (black arrows) at presentation. (b) Cortical union (arrow) four weeks later

A 12-year-old boy was referred to the orthopedic department with a suspected malignancy in the left femur. He had had exertional pain in the left thigh for several months. After a fall four days before the visit, pain became constant and sharper and especially severe with weight-bearing. On clinical examination, no palpable mass was detected but only a tender point over the distal thigh. In the distal femoral diaphysis, radiography revealed a large eccentric heterogeneous area with somewhat dulled osteosclerotic margins oriented along the axis of the femur consistent with the diagnosis of an NOF. Surprisingly, the overlying periosteum showed atypical interrupted lamellar reaction over a short distance of the near cortex and longer non-interrupted periosteal reaction over the far cortex that gave the lesion a somewhat sinister appearance (Figure 1a). Careful scrutiny of the radiograph detected the cause for the periosteal reaction to be a hair-like incomplete transverse stress fracture involving two-thirds of the diameter of the diaphysis facing the periosteal gap and propagating through the pre-existing NOF (Figure 1a). The patient was put on crutches with touchdown weight-bearing in a long-leg brace. Two weeks later, the pain had diminished and a new layer of periosteal bone was deposited; after four weeks, the patient was completely pain-free, there was advanced periosteal callus formation with a cortical union and a gradual return to activity was initiated (Figure 1b).

## Discussion

In the current literature, NOF has been reported as the most common metaphyseal benign fibrous lesion of the long bones. Usually, it is asymptomatic and incidentally found on radiography [5,6]. It typically features sharp osteosclerotic margins around a lobulated radiolucent sub/intracortical area. Simultaneous existence of this entity and a stress fracture in children and adolescents may predispose to radiological misinterpretation [1].

A stress fracture is usually transverse and could be detected on plain radiographs; however, CT reveals it better, especially in cases of a longitudinal fracture line that could be difficult to establish on a radiograph [7]. CT also presents details in the bone morphology and abnormality in cases of NOF [6].

According to Easley and Kneisl (1997), lesions affecting more than 50% of the bone diameter could predispose to fracture [8]. It should be pointed out that a femoral pathological fracture on a pre-existing NOF is rare [9]. In our case, the pathological fracture could be accepted as an insufficiency fracture.

In cases of an interrupted lamellar periosteal reaction that is generally viewed as a worrisome radiographic feature, as in the presented case, radiographic findings could mimic a bone malignancy [2]. However, a single layer of periosteal new bone formation is typical of a stress fracture and a malignancy could be excluded due to the lack of cortical destruction or tumor extension in the surrounding soft tissues [1]. An infection could also be considered in the differential diagnosis but the exertional nature of pain of several months duration along with the normal laboratory findings ruled out this possibility. Radiographic evidence of advancing bone healing could also help the diagnosis ex juvantibus. In the majority of patients, the fractures are treated non-operatively [8].

## Conclusions

In conclusion, we describe a rare case of a 12-year-old boy with a transverse stress fracture on a pre-existing NOF. With this case, we aim to point out that such a constellation in a child with local pain, together with discordant radiographic features, may be mistaken for a bone sarcoma. Careful history-taking and precise analysis of radiographic data could help avoid misdiagnoses. Even the mildest radiographic findings suggestive of a malignancy should be scrutinized and never overlooked. In case a satisfactory explanation for each of them cannot be found, additional imaging studies are warranted. Insufficiency fractures need serial radiographs to establish fracture healing.
